# *Acta Crystallographica Section B* welcomes three new Co-editors

**DOI:** 10.1107/S2052520626000685

**Published:** 2026-02-01

**Authors:** Louise Dawe, Andrzej Katrusiak, Ashwini Nangia, Amanda Berry

**Affiliations:** a Department of Chemistry and Biochemistry Wilfrid Laurier University 75 University Ave W Waterloo Ontario N2L 3C5 Canada; b Department of Materials Chemistry, Faculty of Chemistry, Adam Mickiewicz University, Poznań, 61-614, Poland; c UPES University of Tomorrow, Dehradun, India; d International Union of Crystallography, Chester, CH1 2HU, United Kingdom

**Keywords:** Co-editors, structural science, crystal engineering, materials research, crystal growth

## Abstract

The newest three members of the Editorial Board of *Acta Crystallographica Section B* are introduced.

We welcome three new Co-editors to the Editorial Board of *Acta Crystallographica Section B*.

Professor Nan Zhang ▸ of Xi’an Jiaotong University, China, is a prominent researcher in materials science, particularly ferroelectrics and electronic materials. She is also a member of the IUCr Commission on Crystallography of Materials. Her research interests also include single-crystal diffraction, total scattering and correlated disorders in perovskites.

Professor Dr Martin U. Piepenbring (formerly Martin U. Schmidt) ▸ is a faculty member of Goethe University in Frankfurt am Main, Germany. His research interests focus on method developments for crystal structure determination of crystal powders and nanocrystalline organic compounds, especially the polymorphism of organic pigments and pharmaceuticals, as well as on disorder and diffuse scattering. With Klaus Hunger, he authored the book *Industrial Organic Pigments, Production, Crystal Structures, Applications* (four editions). He is an expert in crystal modelling, especially on disordered organic compounds, and was a participant in the blind tests of crystal-structure predictions. He has contributed more than 110 research articles and abstracts to the IUCr journals. He is a devoted teacher, as exemplified by his frequent lectures at the Erice Crystallographic Courses.

Dr Kamil Dziubek ▸ is a researcher at the Department of Mineralogy and Crystallography at the University of Vienna, Austria, and is the Chair of the IUCr Commission on High Pressure. He has published extensively in the IUCr journals. His research interests include high pressure, chemical crystallography, crystal growth, X-ray diffraction, optical spectroscopies and metadata deposition. He is a dedicated teacher on university courses and on courses for new emerging crystallographic groups; in 2022, he co-directed the 4th High-Pressure Crystallography Course in Erice. He also participated in the organization of several IUCr Commission on High Pressure Workshops and before that in a few of the long series of High-Pressure Frolic Goats Workshops in Poznań.

This expansion will ensure we continue to cover the full spectrum of topics relevant to our readers, while also addressing important new and emerging areas within the field.

## Figures and Tables

**Figure 1:**
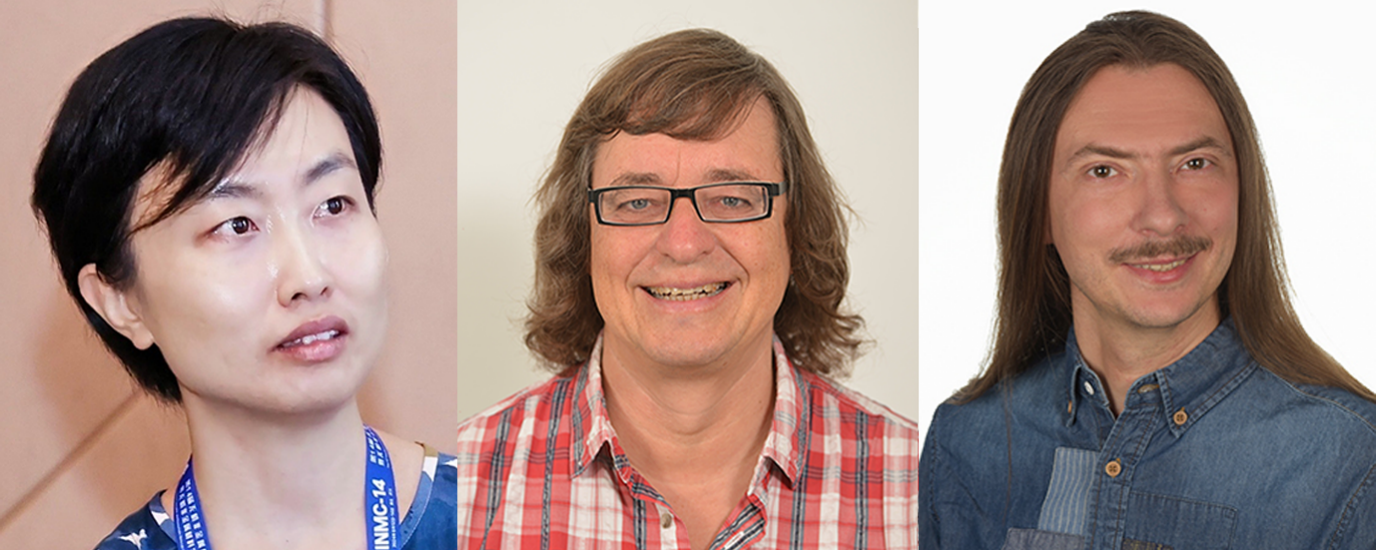
Our new Co-editors: (left to right) Nan Zhang, Martin Piepenbring and Kamil Dziubek.

